# Dementia and metabolic syndrome: a bibliometric analysis

**DOI:** 10.3389/fnagi.2024.1400589

**Published:** 2024-06-12

**Authors:** Tao Peng, Yifan Yang, Jingying Ma, Peili Xu, Xinchun Xie, Nan Hu, Yongmei Yan

**Affiliations:** ^1^First Clinical Medical College, Shaanxi University of Chinese Medicine, Xianyang, Shaanxi, China; ^2^Department of Encephalopathy, Affiliated Hospital of Shaanxi University of Chinese Medicine, Xianyang, Shaanxi, China

**Keywords:** dementia, Alzheimer’s disease, metabolic syndrome, bibliometrics, Web of Science database

## Abstract

**Background:**

Dementia is a progressive neurodegenerative condition, while metabolic syndrome (MetS) is characterized by a combination of metabolic abnormalities such as hypertension, high blood sugar, and obesity. There exists a connection and overlap between the two conditions in certain aspects, and both are influenced to varying degrees by the process of aging. This study presents an overview of the current research landscape regarding dementia and MetS through bibliometric analysis.

**Methods:**

A systematic search was conducted to retrieve relevant literature on dementia and MetS published between 1 January 2000, and 30 November 2023, from the Web of Science Core Collection database. Various bibliometric tools, including VOSviewer, CiteSpace, and the R software package “bibliometrix,” were utilized for analysis.

**Results:**

A total of 717 articles were identified, showing an upward trend in annual publications. Leading contributors included the United States, Italy, and China, with institutions such as the University of California System at the forefront. The *Journal of Alzheimer’s Disease* emerged as the top publisher, while research published in *Neurology* garnered significant citations. Noteworthy authors encompassed Panza, Francesco; Frisardi, Vincenza; and Feldman, Eva L, with Kristine Yaffe being the most cited author (280 citations). Recent studies have focused on themes like “gut microbiota,” “neuroinflammation,” “fatty acids,” and “microglia.”

**Conclusion:**

This bibliometric analysis summarizes the foundational knowledge structure in the realm of dementia and MetS from 2000 to 2023. By highlighting current research frontiers and trending topics, this analysis serves as a valuable reference for researchers in the field.

## 1 Introduction

As the global population continues to grow and life expectancy increases, aging will become a worldwide challenge. According to the World Health Organization, by 2050, the population aged 65 and above is expected to reach 1.5 billion (Zhylkybekova et al., 2023). Aging, accompanied by the inevitable decline in human physiological processes, is unavoidable. With increasing age, the function of various bodily systems gradually decreases, leading to cellular aging, chronic inflammation, and mitochondrial dysfunction, among other physiological changes (Paneni et al., 2017). Additionally, aging is one of the major risk factors for neurodegenerative diseases such as dementia and Alzheimer’s disease. Dementia, a general term for diseases characterized by cognitive impairment, severely affects the daily life, work, and social interactions of patients (Oxbøll et al., 2024). The global incidence of dementia is rapidly increasing, with an expected 75.6 million people affected by 2030 (Zhang et al., 2020), posing a significant economic burden on society. Recent research has pinpointed cellular aging as a critical element in the aging phenomenon, potentially influencing the progression of Alzheimer’s disease through neuroinflammation (Behfar et al., 2022). Studies have unveiled the presence of secretory phenotype proteins associated with cellular aging, such as interleukin-6, insulin-like growth factor binding protein, transforming growth factor-beta, and matrix metalloproteinase-10, in cerebrospinal fluid and blood plasma samples from Alzheimer’s disease patients (Ray et al., 2007; Duits et al., 2015; Whelan et al., 2019). These findings underscore the potentially pivotal role of cellular aging in the onset and progression of Alzheimer’s disease.

Metabolic syndrome (MetS) is a collection of disorders involving abnormalities in lipid and glucose metabolism, including hypertension, hyperlipidemia, glucose irregularities, and obesity (Barzegar-Amini et al., 2021), thereby heightening the vulnerability to cardiovascular diseases. The global prevalence of MetS has been steadily increasing. Current estimates suggest that over one billion individuals worldwide are afflicted with MetS (Saklayen, 2018), with projections indicating a further rise in numbers. Aging is intricately linked to MetS, with their interplay heightening the susceptibility to cardiovascular diseases. Aging represents a gradual decline in physiological functions, where the presence of MetS can negatively impact energy equilibrium, glucose regulation, and blood pressure control as one age, potentially hastening the aging process. Immune system aging and the chronic inflammatory state during aging are key factors that may contribute to the progression of MetS (Veronica and Esther, 2012). Additionally, the association between MetS and dementia remains contentious. While a cohort study conducted in Singapore identified a connection between MetS and an increased risk of dementia (Ng et al., 2016), conflicting conclusions have been drawn in other studies. Consequently, the intersection of aging, dementia, and MetS presents a multifaceted and overlapping constellation, with research on their relationships still inconclusive. Particularly, investigations into the correlation between aging and these two conditions are in the early stages. Hence, conducting a thorough analysis of the developmental trajectories in the realms of dementia and MetS holds substantial significance.

Bibliometrics serves as a method of literature analysis that assesses the publication output and prevailing status of a specific research domain from both quantitative and qualitative standpoints (Ishmuratova et al., 2023). It systematically delineates the developmental trajectories and focal points within the designated domain. Various bibliometric tools have been devised to aid researchers across diverse disciplines in crafting knowledge frameworks. Prominent tools in this domain encompass CiteSpace (Chen, 2006), VOSviewer (van Eck and Waltman, 2010), and the “bibliometrix R” software package (Aria and Cuccurullo, 2017). These tools facilitate bibliometric inquiries from diverse perspectives, inclusive of co-authorship and institutional collaboration analysis, keyword investigation, and citation scrutiny, offering an efficient means to swiftly apprehend research trends and focal areas in a particular domain.

Despite the escalating focus and comprehension regarding MetS, dementia, and age-related disorders, accompanied by a growing body of studies in this realm, there persists an absence of comprehensive bibliometric scrutiny to delineate the research tendencies and focal areas of MetS and dementia. Visualization techniques provide researchers with a more intuitive comprehension of pivotal aspects. Consequently, the amalgamation of bibliometrics and visualization reviews, termed two-level research (Kumar et al., 2021), has become a prevalent approach to bridge this gap. The primary objective of this study is to meticulously analyze the progression of the MetS and dementia research domains, furnishing a holistic insight into present-day trends and research themes for forthcoming investigations.

## 2 Materials and methods

### 2.1 Retrieval strategy

The literature search was conducted on 6 December 2023, utilizing the Web of Science Core Collection (WoSCC) platform,^1^ covering the timeframe from 1 January 2000, to 30 November 2023. The primary search terms included “dementia,” “vascular dementia,” “Alzheimer’s disease,” “Lewy body dementia,” and “metabolic syndrome.” The search criteria targeted titles, author keywords, and abstracts, with document types limited to “articles” and “reviews,” and the language set to “English.” The Topic (TS) module of WoSCC comprises the paper title (TI), abstract (AB), author keywords (DE), and Keywords Plus (ID), which includes additional terms extracted from referenced literature titles. Utilizing the paper title (TI), abstract (AB), and author keywords (DE) for advanced searches enhances result accuracy (Fu et al., 2012). Further details on the search methodologies are found in Supplementary Table 1.

**FIGURE 1:**
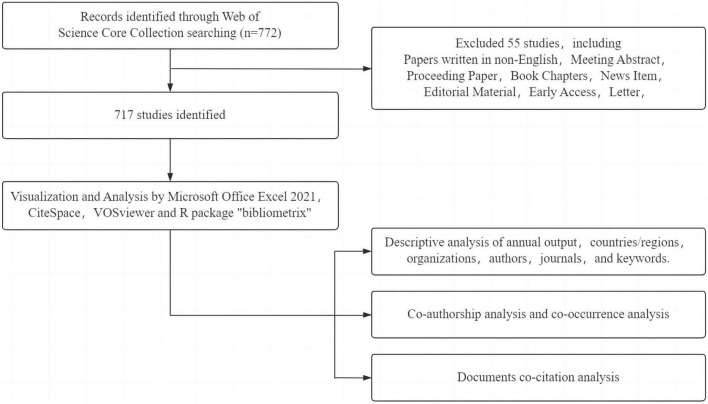
Workflow diagram.

The initial search retrieved 772 documents, comprising 416 articles and 301 reviews. Two researchers independently conducted the literature review and validation process. Upon filtering out literature that did not meet the inclusion criteria, such as meeting abstracts, conference papers, editorial materials, letters, corrections, book chapters, retracted publications, and early access articles, the data was exported in the “full record and citation” format, resulting in 717 documents for analysis. A detailed illustration of the research process is shown in Figure 1.

### 2.2 Data analysis

VOSviewer (version 1.6.20) is a specialized tool for bibliometric analysis, adept at extracting crucial data from a vast array of publications (Pan et al., 2018). Widely utilized for constructing collaborative, co-citation, and co-occurrence networks, this software was instrumental in our study, enabling analyses of countries and institutions, journals and co-cited journals, authors and co-cited authors, as well as keyword co-occurrence. VOSviewer creates visual maps where each node represents a country, institution, journal, or author. Node size and color signify the quantity and category of entities, respectively, while line thickness between nodes reflects the level of collaboration or co-citation.

Developed by Professor Chaomei Chen, CiteSpace (version 6.2.R4) serves as a sophisticated tool for bibliometric analysis and visualization (Synnestvedt et al., 2005). In this study, CiteSpace was utilized to analyze countries and institutions, keyword co-occurrences, clustering, burst analysis, and conducting citation burst analysis on the compiled literature. The visual representation of CiteSpace features nodes representing countries/regions, institutions, keywords, co-cited documents, and co-cited journals. Interconnections between these nodes depict collaborations among countries/regions and institutions, author partnerships, occurrences of keyword co-occurrence, and citations of literature and journals. Central nodes identified by CiteSpace highlight key focus areas within the research field (Wu et al., 2022).

For analyzing sources, keywords, strategic coordinate maps, and developing a global cooperation network from the retrieved articles, the “bibliometrix” R package (version 3.2.1) was utilized, accessible at https://www.bibliometrix.org. Furthermore, publication output trends over the years were visually represented using OriginPro 2023, providing a comprehensive overview of the field’s evolution and collaborative endeavors.

## 3 Results

### 3.1 Publication analysis

**FIGURE 2:**
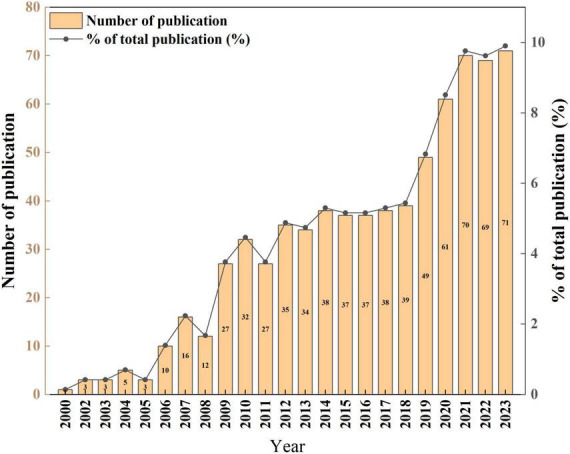
Annual publications trends of dementia and metabolic syndrome.

Figure 2 illustrates a consistent upward trend in research literature concerning MetS and dementia. The analysis of annual publication growth rates delineates the timeline into three distinct phases: Phase I from 2000 to 2008, Phase II from 2009 to 2018, and Phase III from 2019 to 2023. This temporal breakdown, as depicted in Figure 2, unveiled that during Phase I, the field was in its formative stage, with an average annual publication rate of approximately 6.6 papers. Phase II exhibited a notable escalation in research endeavors, evident from an average of 34.4 papers published annually. Notably, Phase III saw a considerable upsurge in research productivity, with an average of about 64 papers per year. Notably, in the last 5 years corresponding to Phase III, there was a sustained and substantial ascent in publications addressing the confluence of MetS and dementia, signifying a growing interest and an expanded research landscape compared to the preceding phases.

### 3.2 Country and region analysis

**FIGURE 3:**
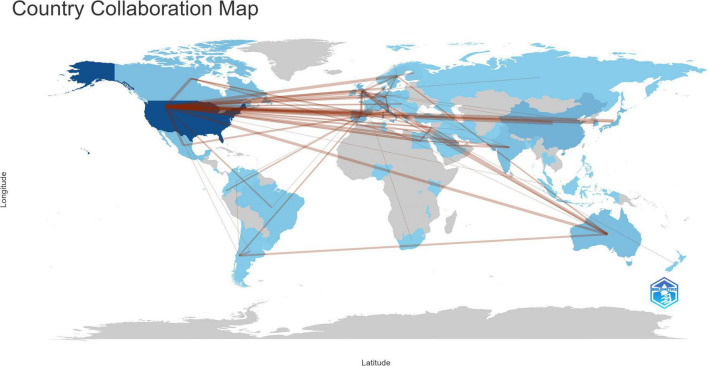
Country collaboration map for dementia and metabolic syndrome.

**FIGURE 4:**
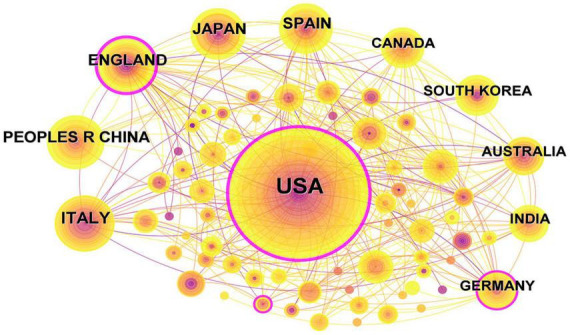
National collaborative map for dementia and metabolic syndrome.

As illustrated in Figure 3, the body of research literature encompassing dementia and MetS emerged from 71 countries and regions. In the figure, the color intensity correlates with the publication volume from each country: darker shades denote higher publication numbers. The foremost contributor in terms of publication volume was the United States, presenting 281 papers, equating to 27.82% of the total, followed by Italy with 64 papers (6.34%), China with 45 papers (4.46%), and the United Kingdom with 44 papers (4.36%). To streamline the analysis, countries with 25 or more publications were accentuated, culminating in the creation of a collaborative network map, as depicted in Figure 4. Each node in this network represents a country or region, with the node size corresponding to its publication volume. Furthermore, the presence of an outer purple ring surrounding the nodes indicates the betweenness centrality of each country, with the ring thickness reflecting the degree of this centrality measure. This metric serves to indicate the pivotal bridging role of a node within the network structure (Brandes, 2001). The network illustrated in Figure 4 elucidated that the United States, the United Kingdom, and Germany exhibited significant betweenness centrality, emphasizing their instrumental roles in fostering research collaborations concerning dementia and MetS.

### 3.3 Institutional analysis

**FIGURE 5:**
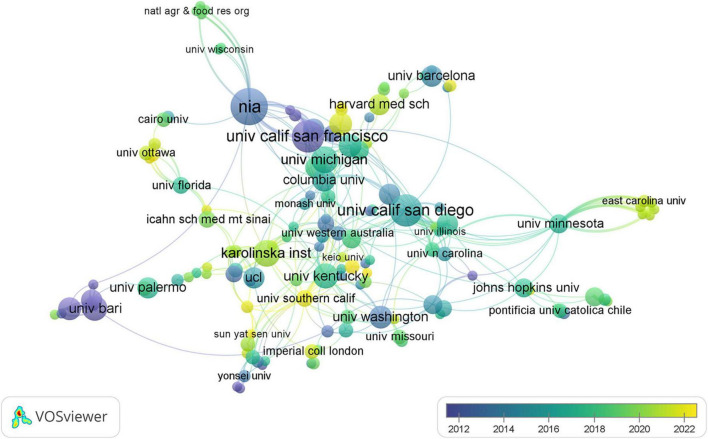
The co-occurrence map of institutions in dementia and metabolic syndrome.

Through a comprehensive examination, it was identified that 402 institutions contributed to the scholarly discourse within this domain, with their collaborative relationships depicted in Figure 5. Within this visualization, each node represents an institution, with node size corresponding to the institution’s publication output. Interconnecting lines denote collaborative endeavors, where the color depth indicates the longevity of the collaboration: deeper hues signify enduring partnerships, while lighter shades denote more recent associations. As detailed in Table 1, among the top 10 institutions by publication volume, eight hailed from the United States, with the remaining establishments located in Sweden and Italy. Noteworthy is the preeminent position held by the University of California System, which boasted the highest publication count (*n* = 38) within this group of institutions, alongside the most substantial centrality score (0.21). This highlights its pivotal role in the collaborative network concerning research on dementia and MetS.

**TABLE 1:** Top 10 institutions in dementia and metabolic syndrome research.

Ranking	Institution	Frequency	Centrality	Country
1	University of California System	38	0.21	USA
2	Veterans Health Administration (VHA)	28	0.04	USA
3	US Department of Veterans Affairs	28	0.04	USA
4	National Institutes of Health (NIH) – USA	18	0.06	USA
5	Harvard University	17	0.11	USA
6	NIH National Institute on Aging (NIA)	13	0.03	USA
7	Universita degli Studi di Bari Aldo Moro	13	0.02	Italy
8	University of California San Francisco	12	0.01	USA
9	Geriatric Research Education and Clinical Center	11	0.01	USA
10	Karolinska Institutet	11	0.05	Sweden

### 3.4 Journal and co-cited journal analysis

This analysis encompassed 717 articles distributed across 377 different journals. Table 2 highlights the top 10 journals by publication count, with the “Journal of Alzheimer’s Disease” leading with 34 papers, followed by the “International Journal of Molecular Sciences” with 24 papers, and “Nutrients” with 12 papers. The average impact factor among these journals stands at 4.97, with half of them positioned within the top quartile (Q1) in their respective fields.

**TABLE 2:** Top 10 journals in publication count.

Rank	Journal	Counts	IF (2023)	JCR
1	Journal of Alzheimer’s Disease	34	4	Q2
2	International Journal of Molecular Sciences	24	5.6	Q1
3	Nutrients	12	5.9	Q1
4	Current Alzheimer Research	9	2.1	Q4
5	Biochimica et Biophysica Acta-Molecular Basis of Disease	7	6.2	Q1
6	Frontiers in Aging Neuroscience	7	4.8	Q2
7	Frontiers in Neuroscience	7	4.3	Q2
8	Molecular Neurobiology	7	5.1	Q2
9	Antioxidants	6	7	Q1
10	Biomedicines	6	4.7	Q1

Table 3 presents the top 10 journals based on their frequency of co-citations. “Neurology” emerges as the most frequently co-cited journal, accumulating 1,492 citations, followed by “Journal of Alzheimer’s Disease” with 1,296 citations, “Journal of Biological Chemistry” with 1,086 citations, and “Proceedings of the National Academy of Sciences of the United States of America” with 1,021 citations, each exceeding a thousand co-citations. Within these leading journals, five hold status as Q1 mainstream publications. Noteworthy is “Nature,” with the highest impact factor of 64.8, underscoring its significant influence within the scientific community.

**TABLE 3:** Top 10 journals in co-citation frequency.

Rank	Co-cited journal	Citations	IF (2023)	JCR
1	Neurology	1,492	10.1	Q1
2	Journal of Alzheimer’s Disease	1,296	4	Q2
3	Journal of Biological Chemistry	1,086	4.8	Q2
4	Proceedings of the National Academy of Sciences of the United States of America	1,021	11.1	Q1
5	PLoS One	940	3.7	Q2
6	Diabetes	914	7.7	Q1
7	Neurobiology of Aging	852	4.2	Q2
8	Diabetes Care	801	16.2	Q1
9	Archives of Neurology	713	/	/
10	Nature	692	64.8	Q1

### 3.5 Author and co-cited author analysis

**FIGURE 6:**
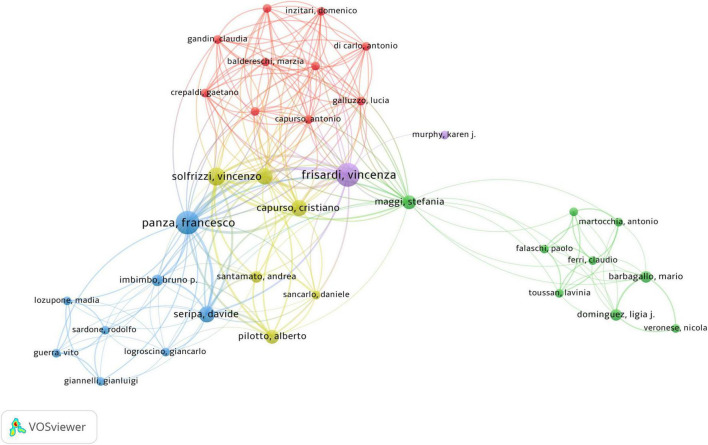
Co-authorship network analysis of dementia and metabolic syndrome.

**FIGURE 7:**
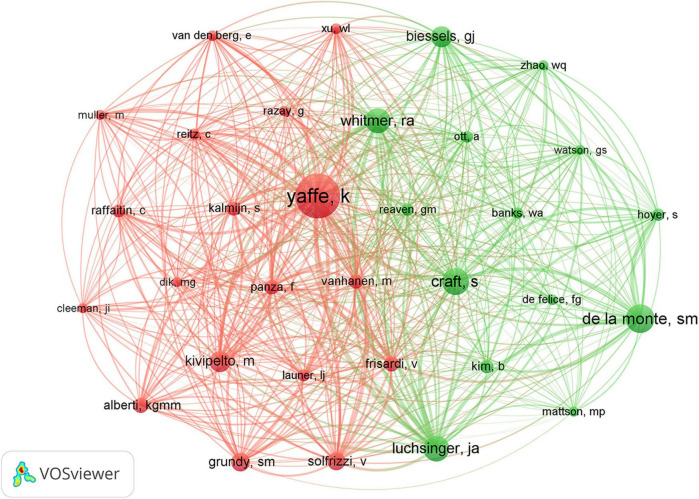
Co-cited authors network on dementia and metabolic syndrome.

The co-authorship network graph in Figure 6 depicted 3,710 authors actively engaged in research pertaining to dementia and MetS. This visual network specifically targeted authors with a collaboration frequency of two or more. Each node in the visualization represented an individual author, with node size correlating to their publication count. Larger nodes denoted a higher publication volume, while the connections between nodes signified co-authorships, whereas denser links indicated more extensive collaborative relationships. Table 4 highlights the top 20 authors making significant contributions to the research on dementia and MetS. These individuals emerged as key figures in the field, often associated within the same research groups, indicative of a robust collaborative network. Notably, Panza, Francesco; Frisardi, Vincenza; and Seripa, Davide from the Orthogeriatric Unit and Center for Diagnosis of Cognitive Disorders and Dementia, Istituto di RicerCa a Carattere Scientifico, have co-authored 23 papers collectively. Feldman, Eva L, ranking third, has authored seven papers, frequently collaborating with Kim, Bhumsoo from the University of Michigan. Cristiano Capurso and Gianluigi Vendemiale, both from the University of Foggia, exemplify effective institutional collaboration. Furthermore, significant contributors include Yaffe, Kristine from the University of California, De La Monte, Suzanne M. from Rhode Island Hospital and the Warren Alpert Medical School of Brown University, and Solfrizzi, Vincenzo from the University of Bari Aldo Moro. In terms of co-cited authors, as depicted in Figure 7, Yaffe, Kristine and De La Monte, Suzanne M. emerged as the most frequently co-cited individuals, highlighting their substantial impact and influence in this research domain.

**TABLE 4:** Top 20 productive authors and co-cited authors.

Ranking	Author	Article	Ranking	Author	Citation
1	Francesco Panza	9	1	Kristine Yaffe	280
2	Vincenza Frisardi	9	2	Suzanne M de la Monte	170
3	Eva L Feldman	7	3	Suzanne Craft	156
4	Kristine Yaffe	6	4	José A Luchsinger	151
5	Suzanne M de la Monte	6	5	Rachel A Whitmer	145
6	Vincenzo Solfrizzi	6	6	Geert Jan Biessels	121
7	Bhumsoo Kim	6	7	Miia Kivipelto	119
8	Cristiano Capurso	5	8	Scott M Grundy	103
9	Gianluigi Vendemiale	5	9	Vincenzo Solfrizzi	101
10	Davide Seripa	5	10	K George M M Alberti	84
11	Gianluigi Vendemiale	5	11	Vincenza Frisardi	83
12	Koji Abe	4	12	Francesco Panza	80
13	Kentaro Deguchi	4	13	S Kalmijn	76
14	Alfonso Diaz	4	14	M Vanhanen	76
15	Jorge Guevara	4	15	Bhumsoo Kim	71
16	Nibaldo C Inestrosa	4	16	C Raffaitin	69
17	Tomoko Kurata	4	17	S Hoyer	65
18	José A Luchsinger	4	18	Gerald M Reaven	65
19	Stefania Maggi	4	19	Lenore J Launer	64
20	Guadalupe Muñoz-Arenas	4	20	A Ott	62

### 3.6 Research hotspots and trends

#### 3.6.1 Co-cited references

**FIGURE 8:**
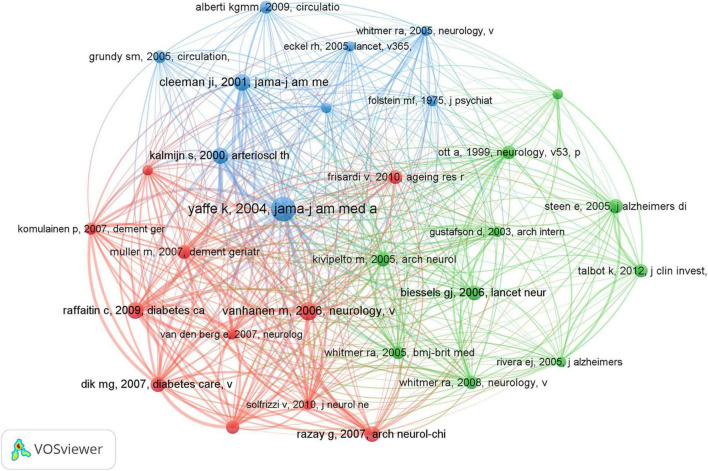
Co-cited network of references in dementia and metabolic syndrome.

Co-cited references are foundational components extensively referenced together in various publications, underpinning specific research domains. In the realm of dementia and MetS studies, a substantial wealth of knowledge has accumulated, with 56,627 co-citations documented over the preceding two decades. Table 5 encapsulates the central research themes associated with the top 10 co-cited references, each amassing 40 or more co-citations. To facilitate a more comprehensive analysis, references with a minimum of 28 co-citations were chosen to construct a co-citation network diagram, visualized in Figure 8. This diagram illuminates the interconnectedness among influential works such as “Yaffe, 2004, JAMA-J Am Med Assoc,” “Vanhanen et al., 2006, Neurology,” and “Raffaitin et al., 2009, Diabetes Care.”

**TABLE 5:** Ten most co-cited references.

Rank	Title	Citations	Publication year	Source
1	The metabolic syndrome, inflammation, and risk of cognitive decline	82	2004	The Journal of the American Medical Association
2	Association of metabolic syndrome with Alzheimer’s disease: a population-based study	58	2006	Neurology
3	Executive summary of the third report of the National Cholesterol Education Program (NCEP) expert panel on detection, evaluation, and treatment of high blood cholesterol in adults (adult treatment panel III)	52	2001	JAMA-Journal of the American Medical Association
4	Metabolic syndrome and risk for incident Alzheimer’s disease or vascular dementia: the Three-City Study	51	2009	Diabetes Care
5	Metabolic cardiovascular syndrome and risk of dementia in Japanese-American elderly men. The Honolulu-Asia aging study	49	2000	Arteriosclerosis Thrombosis and Vascular Biology
6	The metabolic syndrome and Alzheimer’s disease	47	2007	Archives of Neurology
7	Contribution of metabolic syndrome components to cognition in older individuals	45	2007	Diabetes Care
8	Risk of dementia in diabetes mellitus: a systematic review	44	2006	Lancet Neurology
9	Impaired insulin and insulin-like growth factor expression and signaling mechanisms in Alzheimer’s disease–is this type 3 diabetes?	43	2005	Journal of Alzheimer’s Disease
10	Obesity and vascular risk factors at midlife and the risk of dementia and Alzheimer’s disease	42	2005	Archives of Neurology

**FIGURE 9:**
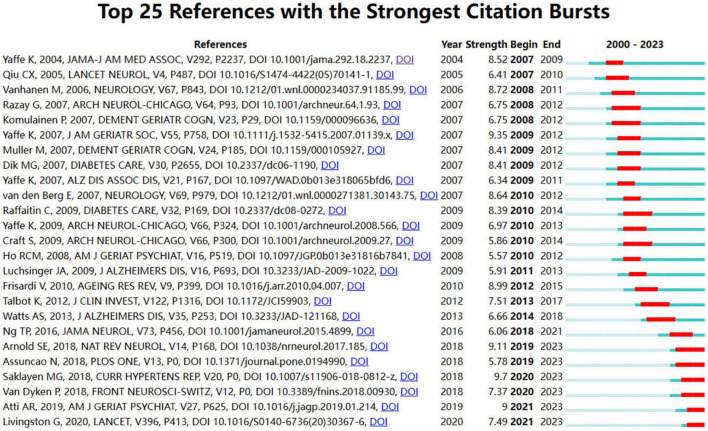
Top 25 burst references of dementia and metabolic syndrome.

The term “citation burst” denotes a surge in citations for specific documents within a defined timeframe, signifying heightened interest and relevance within the research community. Figure 9 outlines the top 25 documents ranked by citation burst, with red bars denoting the duration of each burst. The document exhibiting the highest burst intensity (9.7) is “The Global Epidemic of the Metabolic Syndrome” by Saklayen (2018), which experienced a pronounced citation surge from 2020 to 2023. Closely following is the paper “Metabolic syndrome and cognitive decline in elderly Latinos: findings from the Sacramento Area Latino Study of Aging” by Yaffe et al. (2007), published in the Journal of the American Geriatrics Society, with a substantial citation burst from 2009 to 2012, underscoring its significant impact within the field.

#### 3.6.2 Keyword co-occurrence and cluster analysis

**FIGURE 10:**
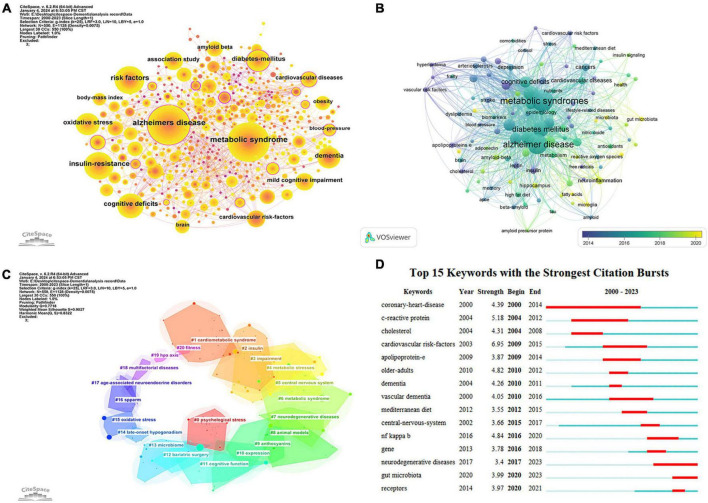
Keyword analysis visualization map. **(A)** Keywords co-occurrence analysis. **(B)** Co-occurrence overlay analysis of author keywords. **(C)** Keywords cluster analysis. **(D)** Keywords burst detection.

Keyword co-occurrence analysis serves as a valuable method to swiftly pinpoint research trends within a specific field. Figure 10A displays a keyword co-occurrence map concerning dementia and MetS research, generated using CiteSpace 6.2.R4. The analysis was enhanced through the application of a g-index (*k* = 25) and the utilization of network pruning techniques such as Pathfinder and Pruning the merged network. This meticulous process culminated in a comprehensive map featuring 550 nodes and 1,128 links, demonstrating a network density of 0.0075. Node size in this visual representation corresponds to the frequency of keyword occurrences, with link thickness indicating the strength of co-occurrence associations between keywords. Noteworthy are keywords encircled by purple lines, denoting high centrality and underscoring their pivotal role and influence within the research network (Zhang et al., 2023). Among the foremost keywords identified were “Alzheimer’s disease” (350 occurrences), “metabolic syndrome” (333 occurrences), and “risk factors” (162 occurrences). In Figure 10B, an overlay visualization of author keywords is presented, where the color gradient from dark blue to light yellow signifies the chronological progression of keyword introductions. Early research predominantly concentrated on terms like “cholesterol,” “leptin,” “blood pressure,” “hyperlipidemia,” and “vascular risk factors.” In contrast, contemporary research has shifted focus toward “gut microbiota,” “neuroinflammation,” “fatty acids,” and “microglia,” signaling the emergence of new research hotspots and thematic directions within the field.

Keyword clustering analysis is employed to assess the distribution and interrelations of studies within a specific field (Pan et al., 2018). In our research, we utilized the Log-Likelihood Ratio (LLR) test algorithm via CiteSpace software. A smaller cluster number indicates a more extensive body of literature, signifying increased research prominence within that particular cluster. Following the keyword clustering analysis, we recognized 21 significant clusters, as illustrated in Figure 10C. The Modularity Q value achieved for this keyword clustering stood at 0.7718 (>0.3), indicative of a robust clustering structure. Moreover, the Mean Silhouette score was calculated at 0.9027 (>0.7), revealing a high degree of coherence among the members of each cluster. These clustering outcomes facilitated the broad categorization of current research trends and focal points into three primary areas: (1) investigation of shared pathological mechanisms, evident in clusters such as #4, #5, #7, #8, #9, #13, and #15; (2) examination of pharmacological treatments, represented in clusters #9 and #16; and (3) analysis of lifestyle factors and preventive strategies, encompassed by clusters #0, #12, #18, and #20.

#### 3.6.3 Keyword burst detection

Keyword burst analysis is a valuable method for identifying significant spikes in the frequency of specific keywords within a brief timeframe, signifying a shared interest among researchers in a specific field (Hu et al., 2022). This technique proves instrumental in monitoring the evolution of trending topics and guiding predictions regarding future research directions. In our investigation, CiteSpace was utilized to pinpoint 15 keywords displaying notable burst patterns. As illustrated in Figure 10D, each horizontal bar corresponds to a year, with red segments denoting periods characterized by intense citation bursts, with the longest burst spanning 14 years. Historically, keywords like “coronary-heart-disease” (2000–2014), “c-reactive protein” (2004–2012), and “cardiovascular risk factors” (2009–2015) commanded prolonged research attention. In a similar vein, “vascular dementia” prominently featured from 2010 to 2016. Conversely, recent keywords such as “neurodegenerative diseases” (2017–2023), “gut microbiota” (2020–2023), and “receptors” (2020–2021) have emerged more recently, indicating a shift toward these areas in contemporary dementia and MetS research.

## 4 Discussion

### 4.1 General information on dementia and MetS research

This study conducts a bibliometric analysis to explore the research trajectory on dementia and MetS over the past two decades. An analysis of annual publication volumes indicates a marked rise in the number of publications, especially notable since 2018. This increasing trend is likely associated with global economic growth, improvements in living standards, and heightened public awareness of health-related issues.

The analysis of collaborative networks among countries, institutions, and authors reveals that the United States is a key player in the field of dementia and MetS research, demonstrating the highest centrality and volume of publications, with Italy also making significant contributions. Remarkably, 80% of the top research institutions in this field are located in the United States. However, from a global perspective, research in this domain is markedly imbalanced, with a concentration in developed countries such as the United States and Europe, while many developing and low-income countries, particularly in regions like Africa and Central and South America, exhibit a severe lack of studies. Despite this, the incidence of dementia and MetS in these regions remains deeply concerning. According to the GBD 2019 Dementia Forecasying Collaborators (2022), the global population affected by dementia is expected to increase from 57 million in 2019 to 153 million by 2050, with the most substantial rise projected in Eastern Sub-Saharan Africa. Here, dementia cases are anticipated to grow by 357%, from approximately 660,000 in 2019 to over 3 million by 2050, with Djibouti, Ethiopia, and South Sudan experiencing the most dramatic increases of 473%, 443%, and 396%, respectively. Similarly, North Africa and the Middle East are predicted to see an increase of 367% in dementia cases, from under 3 million to nearly 14 million, with Qatar, the UAE, and Bahrain facing particularly significant increases of 1,926%, 1,795%, and 1,084%, respectively. Additionally, the prevalence of MetS is notably high in these areas, with rates of 27.65% in South America, 21.27% in Asia, 16.04% in Africa, and 10.47% in Europe (Roomi and Mohammadnezhad, 2019). Given the trends of population growth and aging, both dementia and MetS, whether occurring separately or concomitantly, present significant challenges to socioeconomic development and healthcare systems. Consequently, there is an urgent need for these countries and regions to intensify their research efforts, actively participate in international cooperation and exchanges, and make more substantial contributions to the study and prevention of dementia and MetS.

In terms of individual contributions, Francesco Panza and Vincenza Frisardi stand out as prominent figures with substantial publication counts in this field. On the citation impact front, Kristine Yaffe, Suzanne M. De La Monte, and Suzanne Craft are the top three most highly cited authors, each affiliated with different prestigious American institutions. Although they do not possess the highest number of publications, their profound influence underscores their pivotal roles in advancing research in this area.

Concerning publication venues, the *Journal of Alzheimer’s Disease* leads in terms of article volume, while *Neurology* is the most cited journal. Both journals, which are primarily focused on the treatment and prevention of neurodegenerative diseases, hold high academic prestige and are internationally recognized for their contributions to the field.

### 4.2 Analysis of research hotspots and trends

The analysis of cited references and keywords is crucial for rapidly identifying the distribution and evolution of key themes within the research fields of dementia and MetS (Xu et al., 2023). References or keywords that attract high citation counts indicate significant engagement or prevalent use within a specific timeframe (Sun et al., 2022). Excluding common terms such as “Alzheimer’s disease,” “metabolic syndrome,” and “dementia,” Figure 10A predominantly showcases keywords such as “oxidative stress,” “insulin resistance,” “cognitive impairment,” “body mass index,” and “risk factors.” By integrating keyword clustering and burst detection analyses, the study identifies that research on dementia and MetS may focus on the following areas:

MetS encompasses risk factors such as hypertension, elevated blood sugar, and high cholesterol levels, which significantly increase the likelihood of cardiovascular diseases, stroke, and dementia (Fraade-Blanar et al., 2018; Kumar, 2022; Evans et al., 2023; Junger et al., 2023). There is growing evidence linking MetS with structural brain changes and cognitive decline (Martinelli et al., 2017). Our study underscored the importance of early research keywords like oxidative stress, neuroinflammation, and microglial cells, highlighting their pivotal roles. Oxidative stress and neuroinflammation are recognized as common contributory factors in both dementia and MetS (Popa-Wagner et al., 2018). MetS contributes to the generation of reactive oxygen species, impairs cellular antioxidant defenses, and triggers chronic low-grade inflammation (Buckland et al., 2024). This inflammatory state allows cytokines to breach the blood-brain barrier, leading to neuroinflammation, which facilitates the accumulation and reduced clearance of β-amyloid proteins and the aggregation of tau proteins, thereby exacerbating Alzheimer’s disease pathology. Additionally, oxidative stress is implicated in the formation and accumulation of β-amyloid proteins and the phosphorylation of tau proteins, perpetuating a detrimental cycle (Kim et al., 2015; Rahman et al., 2021). Our findings also emphasized the role of microglial cells and the NF-κB signaling pathway in these processes. Microglial cells, serving as macrophages within the central nervous system, produce inflammatory cytokines and reactive oxygen species that contribute to further neuronal damage (Wang et al., 2021). The NF-κB transcription factor plays a crucial role in regulating the expression of inflammatory markers and adhesion molecules, fostering the recruitment of inflammatory cells (Xu et al., 2015). It also governs the equilibrium between oxidative stress and inflammatory responses by modulating the expression of antioxidant enzymes and NADPH oxidase, thus playing a pivotal role in Alzheimer’s disease progression (Islam, 2017). Recent research has shed light on the connection between gut microbiota and MetS (Mustafa et al., 2022). Fecal microbiota transplantation (FMT) is emerging as a prospective therapeutic avenue. A review by Guzzardi et al. (2023) suggests that FMT can modulate blood glucose and lipid levels by regulating short-chain fatty acids, bile acids, and amino acids. Animal studies indicate that FMT could potentially ameliorate or exacerbate cognitive decline, presenting new treatment prospects for dementia and MetS, albeit necessitating further empirical validation. In conclusion, the interconnected pathogenic mechanisms between MetS and dementia are complex, encompassing elements beyond oxidative stress and neuroinflammation, including genetic and neuroendocrine factors such as dysregulation of the HPA axis (Martocchia et al., 2016).

Our study also revealed the emergence of keywords like “older adults” and “age-associated neuroendocrine disorders,” underscoring the intimate relationship between aging and MetS. Oxidative stress, inflammation, and mitochondrial dysfunction play pivotal roles as crucial mediators of both aging and MetS, with the three factors intricately interacting in this process. Aging contributes to heightened oxidative stress levels, instigating pathophysiological transformations associated with oxidative stress (Veronica and Esther, 2012; Li et al., 2020), while also amplifying inflammation levels and influencing endothelial function through regulatory pathways, cytokine generation, and immune responses (Packard and Libby, 2008). Simultaneously, aging induces disruptions in mitochondrial structure, accumulation of mitochondrial DNA mutations, and a decline in mitochondrial oxidative phosphorylation function (Kraytsberg et al., 2006; Lee et al., 2007; Kowald and Kirkwood, 2011), leading to augmented oxidative stress and excessive apoptosis of neuronal cells. The dysfunctional mitochondria generate a surplus of reactive oxygen species, further exacerbating oxidative stress, damaging proteins and DNA, and sparking lipid peroxidation reactions, thereby hastening the aging process (Watanabe et al., 2021). Furthermore, as individuals age, the advancement of MetS may accelerate aging. MetS has the potential to expedite cell senescence and tissue degradation by impacting the expression of longevity genes, inducing mitochondrial dysfunction, provoking oxidative stress injuries, and affecting overall physiological function (Bonomini et al., 2015). Penninx et al. (2009) demonstrated that compared to individuals without MetS, elderly individuals with MetS exhibited a higher likelihood of physical decline, with hypertension patients notably predisposed to functional decline. The multifaceted adverse effects of aging on MetS and vice versa highlight the intertwined relationship between the two processes, with the presence of MetS further speeding up the aging trajectory. However, despite these profound interconnections, there remains a scarcity of research on comprehensively understanding and exploring the dynamic interplay between aging and MetS, an area deserving increased attention from researchers.

The identification and management of risk factors constitute a fundamental aspect of research in dementia and MetS. Hypertension, obesity, hyperlipidemia, and insulin resistance are recognized as significant pathogenic factors contributing to the development of both MetS and dementia (Fadó et al., 2022). However, these factors are interconnected, complicating the determination of their individual impacts on dementia and their relationships with cognitive decline (Akbaraly et al., 2010). Previous studies suggest diverse responses of MetS components to various dementia subtypes (Rouch et al., 2014). For instance, a strong association is noted between elevated blood sugar, high triglycerides, low HDL levels, Alzheimer’s disease, and MetS (Razay et al., 2007). In contrast, high blood pressure has indicated a negative correlation with the risk of Alzheimer’s disease. However, longitudinal studies have reported conflicting results, indicating that MetS may elevate the risk of vascular dementia, notably linked to high triglyceride levels, diabetes, and overall vascular dementia risk (Raffaitin et al., 2009). Intriguingly, metabolic cardiovascular syndrome appears to elevate the risk of vascular dementia more significantly than Alzheimer’s disease. Moreover, women with MetS face a greater risk of developing Alzheimer’s disease compared to men with MetS (Vanhanen et al., 2006). Discrepancies in these findings may result from variations in research methodologies, statistical methods, follow-up durations, and the presence of confounding variables, necessitating cautious interpretation of the diverse outcomes. Despite these disparities, there is a consensus that individuals with MetS and heightened inflammation levels are at a heightened risk of cognitive impairments relative to those without MetS (Yaffe, 2004). Midlife obesity is associated with an increased risk of dementia and Alzheimer’s disease in later life (Kivipelto et al., 2005), while evidence suggests that hyperinsulinemia and insulin resistance could be crucial factors link MetS and Alzheimer’s disease (Razay et al., 2007). Though definitive conclusions await further detailed investigations, it is indisputable that adopting a healthy lifestyle, including a balanced diet, regular physical activity, efficient stress management, adequate sleep, and appropriate antioxidant supplementation, can mitigate oxidative stress, augment endothelial function, and reduce inflammatory responses (Basu et al., 2023)—essential steps in slowing the aging process and preventing dementia.

Research into drug treatment and preventive strategies plays a vital role in the battle against dementia and MetS. Previous studies have demonstrated that medications commonly utilized for managing MetS or diabetes, including intranasal insulin, metformin, incretin-based therapies, and thiazolidinediones, can also ameliorate some dementia symptoms (Ishmuratova et al., 2023). These drugs have shown potential for enhancing cognitive functions and memory. Recent research has highlighted the promising impact of anthocyanins and selective peroxisome proliferator-activated receptor alpha modulators (SPPARMs) in MetS treatment while reducing cardiovascular risks. Anthocyanins (ANTs), natural pigments belonging to the flavonoid family (He et al., 2019), are abundant in fruits and vegetables like grapes, tomatoes, blueberries, and purple cabbage (Naseri et al., 2018). They offer potential therapeutic benefits in managing MetS and diabetes by enhancing insulin sensitivity, correcting glucose intolerance, normalizing lipid profiles, and reducing hypertension (Naseri et al., 2018; Jiang et al., 2019). Consequently, dietary supplements rich in ANTs could prove beneficial in preventing and managing MetS. Moreover, ANTs have demonstrated potential in dementia treatment (Bergland et al., 2019), improving specific cognitive functions and impacting cholesterol and triglyceride levels (Aarsland et al., 2023). However, confirming these benefits requires larger and more rigorous studies. SPPARMs, exemplified by pemafibrate, represent a new class of drugs showing enhanced efficacy and safety profiles compared to existing therapies. Pemafibrate exhibits promise in modulating lipid profiles by reducing triglycerides, elevating high-density lipoprotein cholesterol levels, mitigating inflammation, and enhancing insulin sensitivity—making it a promising novel treatment for MetS (Fruchart et al., 2021). In addition to pharmaceutical interventions, non-drug strategies such as the Mediterranean diet, physical exercise, and bariatric surgery are gaining recognition. The Mediterranean diet, in particular, is associated with favorable outcomes for both dementia and MetS (Pollicino et al., 2023), attributed to its antioxidant properties. These properties help alleviate oxidative stress on mitochondria and neural cells (Ansari et al., 2023). Components like polyphenols may improve mitochondrial function by influencing genes and proteins related to mitochondria, thereby enhancing the metabolic balance of various tissues and organs and boosting brain energy metabolism. This mechanism likely underlies the diet’s preventive and therapeutic effects on dementia and MetS (Shannon et al., 2023). Encouragingly, researchers are actively exploring drug therapy for Alzheimer’s disease and age-related brain deterioration. Senolytic therapies, like dasatinib and quercetin (P + Q), have demonstrated promising effects in animal models (Bussian et al., 2018; Folk et al., 2019), with related clinical trials underway (Gonzales et al., 2022). In conclusion, although new therapeutic medications and preventive strategies offer innovative paths for addressing dementia and MetS, significant challenges persist, necessitating further research and exploration.

### 4.3 Limitations

While this study marks the first implementation of a bibliometric analysis to unveil trends in dementia and MetS, utmost caution is warranted in interpreting the results and conclusions due to the inherent predictive limitations of this analytical approach. Firstly, the analysis is restricted to trends observed in recent decades, thereby failing to capture the comprehensive breadth of the origins and progression of research regarding dementia and MetS. Second, the data set is limited to the WoSCC, consequently excluding potentially valuable articles from alternative databases. Moreover, the data reviewed in this analysis is exclusively in English, potentially overlooking significant research studies published in other languages. Additionally, the analysis was conducted in November, omitting publications from December and possibly overlooking the latest research findings.

## 5 Conclusion

This study utilizes bibliometric analysis to illuminate the current trends and focal points within the research domains of dementia and MetS. Over the past two decades, a discernible uptick in publication rates has been observed, with notable contributions coming from the United States, Italy, and China. The University of California System emerges as a leading academic institution, generating a substantial volume of publications. In the academic journal realm, the *Journal of Alzheimer’s Disease* and *Neurology* are noteworthy for their significant impact. Kristine Yaffe, representing the United States, is identified as the most influential author in this field. Research endeavors are concentrated on unraveling pathogenesis, exploring drug treatments, and identifying preventive strategies. There exists a growing necessity to investigate the association between MetS and its individual components with dementia. Cross-border collaboration between nations and regions is imperative. Future research endeavors should focus on conducting more extensive, high-quality cohort studies or longitudinal research to deepen insights and facilitate breakthroughs, ultimately enhancing clinical interventions and benefiting a wider patient demographic.

## Data availability statement

The original contributions presented in this study are included in the article/Supplementary material, further inquiries can be directed to the corresponding author.

## Author contributions

TP: Conceptualization, Data curation, Methodology, Software, Visualization, Writing – original draft, Writing – review & editing. YFY: Data curation, Writing – original draft. JM: Data curation, Resources, Writing – review & editing. PX: Data curation, Formal analysis, Methodology, Writing – review & editing. XX: Data curation, Formal analysis, Supervision, Writing – original draft. NH: Data curation, Formal analysis, Visualization, Writing – original draft. YMY: Funding acquisition, Supervision, Writing – review & editing.

## Footnotes

1
https://www.webofscience.com/wos/woscc/basicsearch

## References

[B1] Aarsland D. Khalifa K. Bergland A. K. Soennesyn H. Oppedal K. Holteng L. B. A. (2023). A randomised placebo-controlled study of purified anthocyanins on cognition in individuals at increased risk for dementia. *Am. J. Geriatric Psychiatry* 31 141–151. 10.1016/j.jagp.2022.10.00 36372613

[B2] Akbaraly T. N. Kivimaki M. Shipley M. J. Tabak A. G. Jokela M. Virtanen M. (2010). Metabolic syndrome over 10 years and cognitive functioning in late midlife: the Whitehall II study. *Diabetes Care* 33 84–89. 10.2337/dc09-1218 19837794 PMC2797991

[B3] Ansari U. Wen J. Taguinod I. Nadora D. Nadora D. Lui F. (2023). Exploring dietary approaches in the prevention and management of amyotrophic lateral sclerosis: a literature review. *AIMS Neurosci.* 10 376–387. 10.3934/Neuroscience.2023028 38188002 PMC10767066

[B4] Aria M. Cuccurullo C. J. E. (2017). bibliometrix: an R-tool for comprehensive science mapping analysis. *J. Informetrics* 11 959–975.

[B5] Barzegar-Amini M. Khorramruz F. Ghazizadeh H. Sahebi R. Mohammadi-Bajgyran M. Mohaddes Ardabili H. (2021). Association between serum Vitamin E concentrations and the presence of metabolic syndrome: a population-based cohort study. *Acta biomed.* 92:e2021047. 10.23750/abm.v92i3.9173 34212939 PMC8343740

[B6] Basu T. Sehar U. Malhotra K. Culberson J. Khan H. Morton H. (2023). Healthy brain aging and delayed dementia in Texas rural elderly. *Ageing Res. Rev.* 91:102047. 10.1016/j.arr.2023.102047 37652312 PMC10843417

[B7] Behfar Q. Ramirez Zuniga A. Martino-Adami P. V. (2022). Aging, senescence, and dementia. *J. Prev. Alzheimer’s Dis.* 9 523–531. 10.14283/jpad.2022.42 35841253

[B8] Bergland A. K. Soennesyn H. Dalen I. Rodriguez-Mateos A. Berge R. K. Giil L. M. (2019). Effects of anthocyanin supplementation on serum lipids, glucose, markers of inflammation and cognition in adults with increased risk of dementia - a pilot study. *Front. Genet.* 10:536. 10.3389/fgene.2021.678504 31244884 PMC6581024

[B9] Bonomini F. Rodella L. F. Rezzani R. (2015). Metabolic syndrome, aging and involvement of oxidative stress. *Aging Dis.* 6 109–120. 10.14336/ad.2014.0305 25821639 PMC4365955

[B10] Brandes U. (2001). A faster algorithm for betweeness centrality. *J. Math. Sociol.* 25 163–177.

[B11] Buckland G. Northstone K. Emmett P. M. Taylor C. M. (2024). Associations of childhood diet quality scores with arterial stiffness and carotid artery intima-media thickness in adolescence/early adulthood: findings from the ALSPAC cohort. *Br. J. Nutrition.* 131 720–735. 10.1017/S0007114523002763 38178807 PMC10803818

[B12] Bussian T. J. Aziz A. Meyer C. F. Swenson B. L. van Deursen J. M. Baker D. J. (2018). Clearance of senescent glial cells prevents tau-dependent pathology and cognitive decline. *Nature* 562 578–582. 10.1038/s41586-018-0543-y 30232451 PMC6206507

[B13] Chen C. (2006). CiteSpace II: detecting and visualizing emerging trends and transient patterns in scientific literature. *J. Am. Soc. Information Sci. Technol.* 57 359–377.

[B14] Duits F. H. Hernandez-Guillamon M. Montaner J. Goos J. D. Montañola A. Wattjes M. P. (2015). Matrix metalloproteinases in Alzheimer’s disease and concurrent cerebral microbleeds. *J. Alzheimer’s Dis.* 48 711–720. 10.3233/jad-143186 26402072

[B15] Evans R. Brocklehurst P. Ryan J. Hoare Z. (2023). The impact of different researchers to capture quality of life measures in a dementia randomised controlled trial. *Trials* 24:33. 10.1186/s13063-022-07064-4 36650552 PMC9843939

[B16] Fadó R. Molins A. Rojas R. Casals N. (2022). Feeding the brain: effect of nutrients on cognition, synaptic function, and AMPA receptors. *Nutrients* 14:4137. 10.3390/nu14194137 36235789 PMC9572450

[B17] Folk W. P. Kumari A. Iwasaki T. Pyndiah S. Johnson J. C. Cassimere E. K. (2019). Loss of the tumor suppressor BIN1 enables ATM Ser/Thr kinase activation by the nuclear protein E2F1 and renders cancer cells resistant to cisplatin. *J. Biol. Chem.* 294 5700–5719. 10.1074/jbc.RA118.005699 30733337 PMC6462522

[B18] Fraade-Blanar L. A. Hansen R. N. Chan K. C. G. Sears J. M. Thompson H. J. Crane P. K. (2018). Diagnosed dementia and the risk of motor vehicle crash among older drivers. *Accident Anal. Preven.* 113 47–53. 10.1016/j.aap.2017.12.021 29407668 PMC5869102

[B19] Fruchart J. C. Hermans M. P. Fruchart-Najib J. Kodama T. (2021). Selective peroxisome proliferator-activated receptor alpha modulators (SPPARMα) in the metabolic syndrome: is pemafibrate light at the end of the tunnel? *Curr. Atherosc. Rep.* 23:3. 10.1007/s11883-020-00897-x 33392801 PMC7779417

[B20] Fu H. Z. Wang M. H. Ho Y. S. (2012). The most frequently cited adsorption research articles in the science citation index (Expanded). *J. Colloid. Interface Sci.* 379 148–156. 10.1016/j.jcis.2012.04.051 22608849

[B21] GBD 2019 Dementia Forecasying Collaborators (2022). Estimation of the global prevalence of dementia in 2019 and forecasted prevalence in 2050: an analysis for the Global burden of disease study 2019. *Lancet Public Health* 7 e105–25. 10.1016/S2468-2667(21)00249-8 34998485 PMC8810394

[B22] Gonzales M. M. Garbarino V. R. Marques Zilli E. Petersen R. C. Kirkland J. L. Tchkonia T. (2022). Senolytic Therapy to modulate the progression of Alzheimer’s disease (SToMP-AD): a pilot clinical trial. *J. Prevent. Alzheimer’s Dis.* 9 22–29. 10.14283/jpad.2021.62 35098970 PMC8612719

[B23] Guzzardi M. A. La Rosa F. Iozzo P. (2023). Trust the gut: outcomes of gut microbiota transplant in metabolic and cognitive disorders. *Neurosci. Biobehav. Rev.* 149:105143. 10.1016/j.neubiorev.2023.105143 36990372

[B24] He L. Tang R. Shi X. Wang W. Cao Q. Liu X. (2019). Uncovering anthocyanin biosynthesis related microRNAs and their target genes by small RNA and degradome sequencing in tuberous roots of sweetpotato. *BMC Plant Biol.* 19:232. 10.1186/s12870-019-1790-2 31159725 PMC6547535

[B25] Hu M. Li J. Baker P. N. Tong C. (2022). Revisiting preeclampsia: a metabolic disorder of the placenta. *FEBS J.* 289 336–354. 10.1111/febs.15745 33529475

[B26] Ishmuratova A. N. Abramov M. A. Kuznetsov K. O. Ivanyuta M. V. Shakirova Z. F. Kitapova A. I. (2023). The role of antidiabetic drugs in the treatment of Alzheimer’s disease: systematic review. *Problemy Endokrinologii* 69 73–83. 10.14341/probl13183 37968954 PMC10680548

[B27] Islam M. T. (2017). Oxidative stress and mitochondrial dysfunction-linked neurodegenerative disorders. *Neurol. Res.* 39 73–82. 10.1080/01616412.2016.1251711 27809706

[B28] Jiang X. Li X. Zhu C. Sun J. Tian L. Chen W. (2019). The target cells of anthocyanins in metabolic syndrome. *Crit. Rev. Food Sci. Nutrition* 59 921–946. 10.1080/10408398.2018.1491022 29993258

[B29] Junger A. L. de Sousa Romeiro A. M. Noll M. de Oliveira C. Silveira E. A. (2023). Impact of type, intensity, frequency, duration and volume of physical activity on dementia and mild cognitive impairment in older adults: protocol for a systematic review and meta-analysis. *BMJ open* 13:e074420. 10.1136/bmjopen-2023-074420 38149424 PMC10711853

[B30] Kim G. H. Kim J. E. Rhie S. J. Yoon S. (2015). The role of oxidative stress in neurodegenerative diseases. *Exp. Neurobiol.* 24 325–340. 10.5607/en.2015.24.4.325 26713080 PMC4688332

[B31] Kivipelto M. Ngandu T. Fratiglioni L. Viitanen M. Kåreholt I. Winblad B. (2005). Obesity and vascular risk factors at midlife and the risk of dementia and Alzheimer disease. *Arch. Neurol.* 62 1556–1560. 10.1001/archneur.62.10.1556 16216938

[B32] Kowald A. Kirkwood T. B. (2011). Evolution of the mitochondrial fusion-fission cycle and its role in aging. *Proc. Natl. Acad. Sci. U S A.* 108 10237–10242. 10.1073/pnas.1101604108 21646529 PMC3121810

[B33] Kraytsberg Y. Kudryavtseva E. McKee A. C. Geula C. Kowall N. W. Khrapko K. (2006). Mitochondrial DNA deletions are abundant and cause functional impairment in aged human substantia nigra neurons. *Nat. Genet.* 38 518–520. 10.1038/ng1778 16604072

[B34] Kumar R. Singh S. Sidhu A. S. Pruncu C. I. J. S. (2021). Bibliometric analysis of specific energy consumption (SEC) in machining operations: a sustainable response. *Sustainability* 14:5617.

[B35] Kumar V. H. S. (2022). Cardiovascular morbidities in adults born preterm: getting to the heart of the matter! *Children* 9:1843. 10.3390/children9121843 36553286 PMC9777245

[B36] Lee S. Jeong S. Y. Lim W. C. Kim S. Park Y. Y. Sun X. (2007). Mitochondrial fission and fusion mediators, hFis1 and OPA1, modulate cellular senescence. *J. Biol. Chem.* 282 22977–22983. 10.1074/jbc.M700679200 17545159

[B37] Li J. Zhao S. Lee M. Yin Y. Li J. Zhou Y. (2020). Reliable tumor detection by whole-genome methylation sequencing of cell-free DNA in cerebrospinal fluid of pediatric medulloblastoma. *Sci. Adv.* 6:eabb5427. 10.1126/sciadv.abb5427 33067228 PMC7567591

[B38] Martinelli I. Tomassoni D. Moruzzi M. Traini E. Amenta F. Tayebati S. K. (2017). Obesity and metabolic syndrome affect the cholinergic transmission a nd cognitive functions. *CNS Neurol. Disord. Drug Targets* 16 664–676. 10.2174/1871527316666170428123853 28462694

[B39] Martocchia A. Stefanelli M. Falaschi G. M. Toussan L. Ferri C. Falaschi P. (2016). Recent advances in the role of cortisol and metabolic syndrome in age-related degenerative diseases. *Aging Clin. Exp. Res.* 28 17–23. 10.1007/s40520-015-0353-0 25813987

[B40] Mustafa F. Chopra H. Baig A. A. Avula S. K. Kumari S. Mohanta T. K. (2022). Edible mushrooms as novel myco-therapeutics: effects on lipid level, obesity and BMI. *J. Fungi* 8:211. 10.3390/jof8020211 35205965 PMC8880354

[B41] Naseri R. Farzaei F. Haratipour P. Nabavi S. F. Habtemariam S. Farzaei M. H. (2018). Anthocyanins in the management of metabolic syndrome: a pharmacological and biopharmaceutical review. *Front. Pharmacol.* 9:1310. 10.3389/fphar.2018.01310 30564116 PMC6288909

[B42] Ng T. P. Feng L. Nyunt M. S. Feng L. Gao Q. Lim M. L. (2016). Metabolic syndrome and the risk of mild cognitive impairment and progression to dementia: follow-up of the singapore longitudinal ageing study cohort. *JAMA Neurol.* 73 456–463. 10.1001/jamaneurol.2015.4899 26926205

[B43] Oxbøll A. B. Jørgensen K. Nielsen T. R. Christiansen S. D. Nielsen A. Waldorff F. B. (2024). Diagnostic accuracy of BASIC-Q for detection of cognitive impairment in a primary care setting - a cross-validation study. *BMC Geriatrics* 24:53. 10.1186/s12877-024-04675-1 38212693 PMC10785536

[B44] Packard R. R. Libby P. (2008). Inflammation in atherosclerosis: from vascular biology to biomarker discovery and risk prediction. *Clin. Chem.* 54 24–38. 10.1373/clinchem.2007.097360 18160725

[B45] Pan X. Yan E. Cui M. Hua W. (2018). Examining the usage, citation, and diffusion patterns of bibliometric mapping software: a comparative study of three tools. *J. Informetrics* 12 481–493. 10.1016/j.joi.2018.03.005

[B46] Paneni F. Diaz Cañestro C. Libby P. Lüscher T. F. Camici G. G. (2017). The aging cardiovascular system: understanding it at the cellular and clinical levels. *J. Am. Coll. Cardiol.* 69 1952–1967. 10.1016/j.jacc.2017.01.064 28408026

[B47] Penninx B. W. Nicklas B. J. Newman A. B. Harris T. B. Goodpaster B. H. Satterfield S. (2009). Metabolic syndrome and physical decline in older persons: results from the health, aging and body composition study. *J. Gerontol. Ser. A Biol. Sci. Med. Sci.* 64 96–102. 10.1093/gerona/gln005 19164274 PMC2691185

[B48] Pollicino F. Veronese N. Dominguez L. J. Barbagallo M. (2023). Mediterranean diet and mitochondria: new findings. *Exp. Gerontol.* 176:112165. 10.1016/j.exger.2023.112165 37019345

[B49] Popa-Wagner A. Glavan D. G. Olaru A. Olaru D. G. Margaritescu O. Tica O. (2018). Present status and future challenges of new therapeutic targets in preclinical models of stroke in aged animals with/without comorbidities. *Int. J. Mol. Sci.* 19:356. 10.3390/ijms19020356 29370078 PMC5855578

[B50] Raffaitin C. Gin H. Empana J. P. Helmer C. Berr C. Tzourio C. (2009). Metabolic syndrome and risk for incident Alzheimer’s disease or vascular dementia: the three-city study. *Diabetes Care* 32 169–174. 10.2337/dc08-0272 18945929 PMC2606808

[B51] Rahman M. H. Bajgai J. Fadriquela A. Sharma S. Trinh T. T. Akter R. (2021). Therapeutic potential of natural products in treating neurodegenerative disorders and their future prospects and challenges. *Molecules* 26:5327. 10.3390/molecules26175327 34500759 PMC8433718

[B52] Ray S. Britschgi M. Herbert C. Takeda-Uchimura Y. Boxer A. Blennow K. (2007). Classification and prediction of clinical Alzheimer’s diagnosis based on plasma signaling proteins. *Nat. Med.* 13 1359–1362. 10.1038/nm1653 17934472

[B53] Razay G. Vreugdenhil A. Wilcock G. (2007). The metabolic syndrome and Alzheimer disease. *Arch. Neurol.* 64 93–96. 10.1001/archneur.64.1.93 17210814

[B54] Roomi M. A. Mohammadnezhad M. (2019). Prevalence of metabolic syndrome among apparently healthy workforce. *J. Ayub Med. Coll. Abbottabad* 31 252–254. 31094127

[B55] Rouch I. Trombert B. Kossowsky M. P. Laurent B. Celle S. Ntougou Assoumou G. (2014). Metabolic syndrome is associated with poor memory and executive performance in elderly community residents: the PROOF study. *Am. J. Geriatric Psychiatry* 22 1096–1104. 10.1016/j.jagp.2014.01.005 24534523

[B56] Saklayen M. G. (2018). The global epidemic of the metabolic syndrome. *Curr. Hypertension Rep.* 20:12. 10.1007/s11906-018-0812-z 29480368 PMC5866840

[B57] Shannon O. M. Ranson J. M. Gregory S. Macpherson H. Milte C. Lentjes M. (2023). Mediterranean diet adherence is associated with lower dementia risk, independent of genetic predisposition: findings from the UK Biobank prospective cohort study. *BMC Med.* 21:81. 10.1186/s12916-023-02772-3 36915130 PMC10012551

[B58] Sun H. L. Bai W. Li X. H. Huang H. Cui X. L. Cheung T. (2022). Schizophrenia and inflammation research: a bibliometric analysis. *Front. Immunol.* 13:907851. 10.3389/fimmu.2022.907851 35757702 PMC9219580

[B59] Synnestvedt M. B. Chen C. Holmes J. H. (2005). CiteSpace II: visualization and knowledge discovery in bibliographic databases. *Annu. Symp. Proc. AMIA Symp.* 2005 724–728. 16779135 PMC1560567

[B60] van Eck N. J. Waltman L. (2010). Software survey: VOSviewer, a computer program for bibliometric mapping. *Scientometrics* 84 523–538. 10.1007/s11192-009-0146-3 20585380 PMC2883932

[B61] Vanhanen M. Koivisto K. Moilanen L. Helkala E. L. Hänninen T. Soininen H. (2006). Association of metabolic syndrome with Alzheimer disease: a population-based study. *Neurology* 67 843–847. 10.1212/01.wnl.0000234037.91185.99 16966548

[B62] Veronica G. Esther R. R. (2012). Aging, metabolic syndrome and the heart. *Aging Dis.* 3 269–279. 22724085 PMC3375083

[B63] Wang Y. Ge X. Yu S. Cheng Q. (2021). Achyranthes bidentata polypeptide alleviates neurotoxicity of lipopolysaccharide-activated microglia via PI3K/Akt dependent NOX2/ROS pathway. *Ann. Transl. Med.* 9:1522. 10.21037/atm-21-4027 34790728 PMC8576683

[B64] Watanabe H. Bagarinao E. Maesawa S. Hara K. Kawabata K. Ogura A. (2021). Characteristics of neural network changes in normal aging and early dementia. *Front. Aging Neurosci.* 13:747359. 10.3389/fnagi.2021.747359 34880745 PMC8646086

[B65] Whelan C. D. Mattsson N. Nagle M. W. Vijayaraghavan S. Hyde C. Janelidze S. (2019). Multiplex proteomics identifies novel CSF and plasma biomarkers of early Alzheimer’s disease. *Acta Neuropathol. Commun.* 7:169. 10.1186/s40478-019-0795-2 31694701 PMC6836495

[B66] Wu H. Cheng K. Tong L. Wang Y. Yang W. Sun Z. (2022). Knowledge structure and emerging trends on osteonecrosis of the femoral head: a bibliometric and visualized study. *J. Orthopaedic Surg. Res.* 17:194. 10.1186/s13018-022-03068-7 35346273 PMC8960091

[B67] Xu F. Xia C. Dou L. Huang X. (2023). Knowledge mapping of exosomes in metabolic diseases: a bibliometric analysis (2007-2022). *Front. Endocrinol.* 14:1176430. 10.3389/fendo.2023.1176430 37223047 PMC10200891

[B68] Xu Z. Yoshida T. Wu L. Maiti D. Cebotaru L. Duh E. J. (2015). Transcription factor MEF2C suppresses endothelial cell inflammation via regulation of NF-κB and KLF2. *J. Cell Physiol.* 230 1310–1320. 10.1002/jcp.24870 25474999 PMC4519036

[B69] Yaffe K. (2004). The metabolic syndrome, inflammation, and risk of cognitive decline. *JAMA* 292 2237–2242. 10.1001/jama.292.18.2237 15536110

[B70] Yaffe K. Haan M. Blackwell T. Cherkasova E. Whitmer R. A. West N. (2007). Metabolic syndrome and cognitive decline in elderly latinos: findings from the sacramento area latino study of aging study. *J. Am. Geriatrics Soc.* 55 758–762. 10.1111/j.1532-5415.2007.01139.x 17493197

[B71] Zhang J. Sun P. Zhou C. Zhang X. Ma F. Xu Y. (2020). Regulatory microRNAs and vascular cognitive impairment and dementia. *CNS Neurosci. Therapeutics* 26 1207–1218. 10.1111/cns.13472 33459504 PMC7702235

[B72] Zhang R. Tao Y. Huang J. (2023). The application of MicroRNAs in glaucoma research: a bibliometric and visualized analysis. *Int. J. Mol. Sci.* 24:15377. 10.3390/ijms242015377 37895056 PMC10607922

[B73] Zhylkybekova A. Turlayev A. Grjibovski A. M. Koshmaganbetova G. K. (2023). Measures to support informal care for the older adults in Kazakhstan: a review of the current status. *Front. Public Health* 11:1247684. 10.3389/fpubh.2023.1247684 37670833 PMC10475596

