# Supplementary material for: Dementia and metabolic syndrome: a bibliometric analysis
Source: Front Aging Neurosci. 2024 Jun 12;16:1400589. doi: 10.3389/fnagi.2024.1400589 (PMC11199533; doi:10.3389/fnagi.2024.1400589)
Supplement: Supplementary file 1 [file Data_Sheet_1.docx]

Supplementary Material

Table 1: Details of the Literature Search Strategy

(1) Web of Science (Nov 30, 2023)

| Search | Query | Items found |
| --- | --- | --- |
| #1 | ((TI=("Metabolic syndrome" OR "Insulin resistance syndrome" OR "MetS" OR "Dysmetabolic Syndrome" OR "Cardiometabolic Syndrome" OR "Metabolic X Syndrome" OR "Syndrome X" OR "Reaven's Syndrome" OR "metabolic syndrome X" OR "dysmetabolic syndrome X" OR "insulin resistance syndrome X" OR "Reaven Syndrome" OR "Metabolic Cardiovascular Syndrome")) OR AB=("Metabolic syndrome" OR "Insulin resistance syndrome" OR "MetS" OR "Dysmetabolic Syndrome" OR "Cardiometabolic Syndrome" OR "Metabolic X Syndrome" OR "Syndrome X" OR "Reaven's Syndrome" OR "metabolic syndrome X" OR "dysmetabolic syndrome X" OR "insulin resistance syndrome X" OR "Reaven Syndrome" OR "Metabolic Cardiovascular Syndrome")) OR AK=("Metabolic syndrome" OR "Insulin resistance syndrome" OR "MetS" OR "Dysmetabolic Syndrome" OR "Cardiometabolic Syndrome" OR "Metabolic X Syndrome" OR "Syndrome X" OR "Reaven's Syndrome" OR "metabolic syndrome X" OR "dysmetabolic syndrome X" OR "insulin resistance syndrome X" OR "Reaven Syndrome" OR "Metabolic Cardiovascular Syndrome") | 76,032 |
| #2 | ((TI=("Dementia" OR "Alzheimer" OR "alzheimer dementia" OR "dementia Alzheimer type" OR "sclerosis Alzheimer" OR "alzheimer syndrome" OR "Vascular Dementia" OR "Subcortical Vascular Dementia" OR "infarct dementia" OR "vascular cognitive impairment" OR "Multi-Infarct Dementia" OR "Senile Dementia" OR "Dementia with Lewy Bodies" OR "Lewy Body Parkinson's Disease" OR "Frontotemporal dementia" OR "Frontotemporal lobar degeneration" OR "Parkinson disease with dementia")) OR AB=("Dementia" OR "Alzheimer" OR "alzheimer dementia" OR "dementia Alzheimer type" OR "sclerosis Alzheimer" OR "alzheimer syndrome" OR "Vascular Dementia" OR "Subcortical Vascular Dementia" OR "infarct dementia" OR "vascular cognitive impairment" OR "Multi-Infarct Dementia" OR "Senile Dementia" OR "Dementia with Lewy Bodies" OR "Lewy Body Parkinson's Disease" OR "Frontotemporal dementia" OR "Frontotemporal lobar degeneration" OR "Parkinson disease with dementia")) OR AK=("Dementia" OR "Alzheimer" OR "alzheimer dementia" OR "dementia Alzheimer type" OR "sclerosis Alzheimer" OR "alzheimer syndrome" OR "Vascular Dementia" OR "Subcortical Vascular Dementia" OR "infarct dementia" OR "vascular cognitive impairment" OR "Multi-Infarct Dementia" OR "Senile Dementia" OR "Dementia with Lewy Bodies" OR "Lewy Body Parkinson's Disease" OR "Frontotemporal dementia" OR "Frontotemporal lobar degeneration" OR "Parkinson disease with dementia") | 253,831 |
| #3 | #1 AND #2 | 772 |
| #4 | #1AND #2 and Article or Review Article (Document Types) and Science Citation Index Expanded (SCI-EXPANDED) (Web of Science Index) and English (Languages) | 717 |
|  |  |  |
|  |  |  |
